# Predictors and a Nomogram for Parenchymal Hematoma After Endovascular Thrombectomy in Large Core Ischemic Stroke

**DOI:** 10.1002/cns.71087

**Published:** 2026-08-13

**Authors:** Jiamin Li, Zixin Wang, Xinyi Wang, Boyi Yuan, Yun Chen, Wan Wang, Jiameng Li, Jiapeng Zhao, Jinming Sheng, Qingfeng Ma

**Affiliations:** ^1^ Department of Neurology Xuanwu Hospital, Capital Medical University Beijing China; ^2^ National Center for Neurological Disorders Beijing China

**Keywords:** endovascular thrombectomy, large core infarction, parenchymal hematoma, risk prediction model, stroke

## Abstract

**Objective:**

We sought to establish a nomogram with internal validation to predict parenchymal hematoma (PH) following endovascular thrombectomy (EVT) in patients with large core infarcts.

**Methods:**

We retrospectively enrolled 133 consecutive patients with large core anterior circulation infarctions receiving EVT from March 2018 to December 2024. Following univariable analysis (*p* < 0.1), LASSO and multivariable logistic regression were utilized to investigate predictive factors and establish a PH prediction nomogram. Model performance was assessed via ROC, calibration, decision, and clinical impact curves, with 1000‐bootstrap internal validation. Subgroup analyses were stratified by age, baseline ASPECTS, and prior intravenous thrombolysis (IVT).

**Results:**

PH occurred in 26 patients (19.5%). Baseline NIHSS score (OR 1.109, 95% CI 1.017–1.221; *p* = 0.023), monocytes (OR 0.002, 95% CI 9.74 × 10^−6^–0.174; *p* = 0.012), platelets (OR 0.989, 95% CI 0.978–0.998; *p* = 0.028), post‐thrombectomy fasting blood glucose (FBG) (OR 1.326, 95% CI 1.121–1.616; *p* = 0.002), and number of passes (OR 2.312, 95% CI 1.443–3.990; *p* = 0.001) were significantly associated with PH and incorporated to construct the nomogram. The model showed good discrimination (AUC = 0.876; adjusted AUC = 0.844) and acceptable calibration (Hosmer‐Lemeshow *p* = 0.928; adjusted Brier score = 0.128). Subgroup analyses indicated a significant interaction between number of passes and IVT (P for interaction = 0.020): increased passes elevated PH risk only in patients without bridging IVT (OR 2.055, 95% CI 1.318–3.204; *p* = 0.001).

**Conclusions:**

This nomogram provides a robust tool for predicting post‐thrombectomy PH in large core infarctions. The interaction between IVT and the number of passes potentially indicates minimizing unnecessary passes in direct thrombectomy. Multicenter prospective validation is warranted.

## Graphical abstract

This study developed and internally validated a nomogram incorporating baseline NIHSS score, monocytes, platelets, post‐thrombectomy fasting blood glucose, and number of passes to predict parenchymal hematoma after EVT in large core infarctions. A pass‐by‐IVT interaction suggests minimizing unnecessary passes during direct thrombectomy.

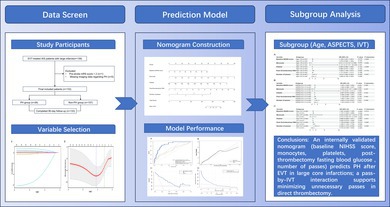

## 1 Introduction

Large core ischemic stroke accounts for approximately 11%–13% of patients with anterior circulation large vessel occlusion who present during the 6–24 h time window [1, 2]. Accumulating evidence from recent randomized controlled trials (RCTs) has revealed the functional benefit of EVT over standard medical treatment alone among patients with large core infarctions [3, 4, 5, 6, 7]. Nevertheless, one of the most significant concerns is hemorrhagic transformation (HT) that occurs following EVT. A meta‐analysis of six RCTs comprising 1887 patients showed that EVT is linked to elevated symptomatic intracranial hemorrhage (sICH) risk [8]. Among the subtypes of HT, parenchymal hematoma (PH) is the most severe complication that significantly raises 90‐day mortality [9]. Therefore, identifying individuals with a higher sensitivity to PH at an early stage may help clinicians implement appropriate management strategies and improve clinical outcomes.

Several predictors for PH after EVT in large core ischemic stroke have been reported, including alcohol use, number of thrombectomy attempts, and local sedative anesthesia [9, 10]. However, the predictors of PH remain poorly characterized across critical clinical subsets, such as elderly patients, those receiving intravenous thrombolysis before EVT, or individuals with extensive ischemic cores (Alberta Stroke Program Early CT Score [ASPECTS] 0–2). Furthermore, an integrated, clinically practical prediction model for individualized risk assessment in the large‐core population is currently lacking.

This study sought to identify independent clinical predictors of PH after EVT among patients with large core infarctions and constructed a logistic regression–derived nomogram to estimate PH risk. This model may assist in early stratification of patients at high risk for PH and support clinical management decisions. Additionally, we performed preliminary analyses to determine whether the associations between these predictors and PH were modified by age, ASPECTS, or intravenous thrombolysis.

## 2 Methods

### 2.1 Study Design and Participants

The observational study retrospectively included consecutive patients with large core anterior circulation infarctions receiving EVT at Xuanwu Hospital, Capital Medical University during the period of March 2018 to December 2024. Eligibility for the study required: (1) age ≥ 18 years; (2) acute ischemic stroke in the anterior circulation; (3) occlusion of the internal carotid artery (ICA) or the M1/M2 segments of the middle cerebral artery (MCA); (4) evidence of a large core infarction on baseline non‐contrast computed tomography (NCCT), defined as an ASPECTS of 0–5; and (5) receipt of EVT. Exclusion criteria were: (1) lack of follow‐up CT or MRI within 48 h or upon neurological deterioration; (2) pre‐event modified Rankin Scale (mRS) score > 2.

### 2.2 Data Acquisition

Baseline clinical data, imaging findings, and procedural details were retrospectively reviewed and recorded. The collected clinical information comprised demographic characteristics, vascular risk factors, medical history, admission blood pressure, baseline National Institutes of Health Stroke Scale (NIHSS) scores, and TOAST‐defined stroke subtypes. Imaging assessments included baseline ASPECTS and occlusion location. Treatment‐related variables consisted of intravenous thrombolysis use, procedural workflow parameters, and EVT‐related characteristics such as thrombectomy strategy, number of passes, and successful reperfusion.

Laboratory parameters obtained at baseline included leukocyte‐related parameters, neutrophil‐to‐lymphocyte ratio (NLR), platelet count, albumin, prealbumin, lipid profile, uric acid, urea, creatinine, fibrinogen, and D‐dimer. In addition, blood glucose data were collected, including random blood glucose (RBG) at admission and fasting blood glucose (FBG) within 24 h after thrombectomy.

Vascular imaging data from CT angiography (CTA) and digital subtraction angiography (DSA) were reviewed to determine the occluded arterial segments. The final angiographic reperfusion status was graded based on the modified Thrombolysis in Cerebral Infarction (mTICI) classification, with successful reperfusion determined by achieving mTICI grade 2b–3. We applied ASPECTS to quantify the degree of ischemia alterations in the anterior circulation.

### 2.3 Classification of HT

Hemorrhagic transformation was determined by neuroimaging performed 24–48 h after EVT or earlier if neurological deterioration occurred. HT was subsequently categorized by the European Cooperative Acute Stroke Study II (ECASS II) definitions. Two independent radiologists blinded to the participants' baseline clinical data, procedural records, and laboratory results read all follow‐up CT/MRI images. Interpretations were discussed among the readers and consensus was reached, adjudicated if necessary by a senior radiologist. According to the ECASS II criteria, Hemorrhagic infarction type 1 (HI1) was characterized by small petechial hemorrhages without mass effect, whereas hemorrhagic infarction type 2 (HI2) was characterized by confluent petechiae without mass effect [11]. PH1 was described as a hematoma involving ≤ 30% of the infarcted area with mild mass effect, while PH2 was described as a hematoma involving > 30% of the infarcted area with substantial mass effect [11]. The PH group comprised subtypes PH1 and PH2, while patients with punctate hemorrhages (HI1 and HI2) within the ischemic area were categorized into the non‐PH group along with those without hemorrhagic complications.

### 2.4 Study Outcomes

At the 90‐day follow‐up, clinical outcomes consisted of the ordinal modified Rankin Scale (mRS) score and dichotomized functional prognosis, including functional independence defined as mRS 0–2, independent ambulation defined as mRS 0–3, as well as mortality. The 90‐day mRS score was assessed by experienced neurologists via structured telephone follow‐up.

### 2.5 Statistical Analysis

Median imputation was applied to variables with missing values < 5%. Continuous variables were displayed as median with interquartile range (IQR), whereas categorical variables were displayed as frequencies with percentages. Differences between groups were assessed with the Mann–Whitney *U* test for continuous measurements and either Pearson *χ* ^2^ test or Fisher's exact test for categorical comparisons.

To mitigate multicollinearity and prevent overfitting, variables showing *p* < 0.1 in univariable analyses were further evaluated by least absolute shrinkage and selection operator (LASSO) regression using 5‐fold cross‐validation. The variables retained at the optimal λ.min value were then entered into a multivariable logistic regression model, and those predictors independently associated with PH were utilized to develop the nomogram.

Subsequently, we assessed the PH prediction model. Model discrimination was measured by the area under the receiver operating characteristic curve (AUC). Calibration was examined using calibration curves, the Hosmer–Lemeshow goodness‐of‐fit test, and the Brier score. Clinical applicability was assessed by decision curve analysis (DCA) to quantify the net benefit at varying threshold probabilities. Furthermore, the clinical impact curve (CIC) was displayed to visually assess the model's practical usefulness by comparing the estimated number of individuals at high risk with the corresponding actual number of PH events at different threshold levels. Finally, the nomogram was internally validated by 1000 bootstrap resamples to assess the robustness.

Subgroups for age (≥ 70 vs. < 70 years), baseline ASPECTS (0–2 vs. 3–5), and pre‐thrombectomy intravenous thrombolysis were analyzed. We performed univariable comparisons and univariable logistic regression in each subgroup. Effect modification was subsequently evaluated in the overall cohort using unadjusted logistic regression models that included the predictor, the corresponding stratifying variable, and their multiplicative interaction term. No additional covariates were included in the interaction models because of the restricted sample size and limited event frequency.

Furthermore, we employed multivariable logistic regression analyses to investigate the association of PH with other clinical outcomes. PH was set as the primary independent variable, while 90‐day functional independence (mRS 0–2), 90‐day independent ambulation (mRS 0–3), and 90‐day mortality were set as the dependent variables in separate models. These models included prespecified clinically meaningful variables according to prior evidence and expert knowledge, involving age, sex, baseline NIHSS score, baseline ASPECTS, number of passes, and successful reperfusion. Effect estimates were quantified as adjusted ORs with their corresponding 95% CIs.

Data analyses were carried out in SPSS version 27.0 and R version 4.5.3. Statistical tests were interpreted using a two‐sided *p* level of 0.05.

## 3 Results

### 3.1 Baseline Characteristics of the Study Population

**FIGURE 1:**
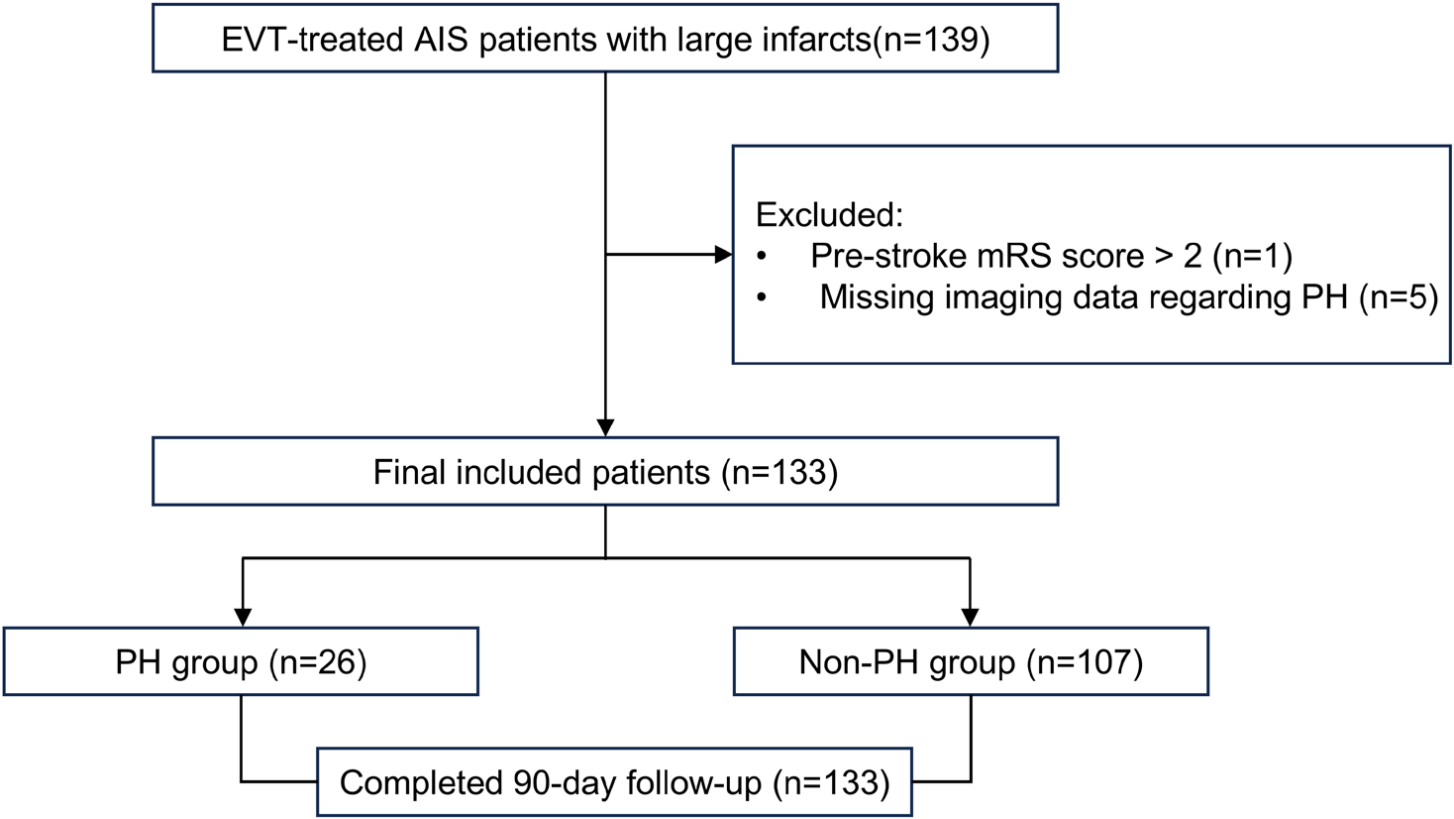
Study flowchart of participant enrollment.

Between March 2018 and December 2024, 139 AIS patients with anterior circulation large‐to‐medium vessel occlusion who received EVT and had baseline ASPECTS ≤ 5 were screened for eligibility. Six patients were excluded, including one with a pre‐event mRS > 2 and five lacking follow‐up neuroimaging for hemorrhage evaluation. Ultimately, 133 eligible patients were retained for the final analysis (Figure 1), comprising 87 males (65.4%). The median age of these patients in the study was 65 (54, 71) years, with baseline NIHSS score of 16 (12, 19) and ASPECTS of 4 (2, 5). 41 patients (30.8%) underwent bridging intravenous thrombolysis (IVT) before EVT. Patients received a median of 2 passes (1, 3), and successful reperfusion was achieved in 124 cases (93.2%). The detailed baseline data are presented in Table 1.

**TABLE 1:** Characteristics of patients in the PH group and the non‐PH group.

Variable	Total (*n* = 133)	PH (*n* = 26)	Non‐PH (*n* = 107)	*p*
Age, years, median (IQR)	65 (54, 71)	65 (58, 73)	64 (53, 71)	0.222
Male sex, *n* (%)	87 (65.4)	16 (61.5)	71 (66.4)	0.643
Prior ischemic stroke, *n* (%)	26 (19.5)	5 (19.2)	21 (19.6)	0.964
Hypertension, *n* (%)	96 (72.2)	20 (76.9)	76 (71.0)	0.547
Diabetes, *n* (%)	35 (26.3)	7 (26.9)	28 (26.2)	0.938
Hyperlipidemia, *n* (%)	68 (51.1)	13 (50.0)	55 (51.4)	0.898
Atrial fibrillation, *n* (%)	44 (33.1)	9 (34.6)	35 (32.7)	0.853
Coronary artery disease, *n* (%)	40 (30.1)	12 (46.2)	28 (26.2)	**0.046** *
Prior use of antiplatelet agents, *n* (%)	36 (27.1)	8 (30.8)	28 (26.2)	0.636
Prior use of anticoagulants, *n* (%)	11 (8.3)	2 (7.7)	9 (8.4)	1.000
Smoke, *n* (%)	62 (46.6)	11 (42.3)	51 (47.7)	0.623
Alcohol, *n* (%)	53 (39.8)	8 (30.8)	45 (42.1)	0.292
Admission SBP, median (IQR), mm Hg	150 (135, 168)	161 (132, 175)	150 (136, 165)	0.335
Admission DBP, median (IQR), mm Hg	84 (76, 94)	89 (77, 96)	82 (76, 94)	0.350
Baseline NIHSS score, median (IQR)	16 (12, 19)	18 (14, 22)	15 (12, 18)	**0.026** *
ASPECTS, median (IQR)	4 (2, 5)	3 (0, 4)	4 (2, 5)	**0.007** **
TOAST classification, *n* (%)				
Large artery atherosclerosis	72 (54.1)	16 (61.5)	56 (52.3)	0.692
Cardioembolism	54 (40.6)	9 (34.6)	45 (42.1)	
Undetermined or other	7 (5.3)	1 (3.8)	6 (5.6)	
Occlusion site, *n* (%)				
ICA	70 (52.6)	16 (61.5)	54 (50.5)	0.501
M1	49 (36.8)	7 (26.9)	42 (39.3)	
M2	14 (10.5)	3 (11.5)	11 (10.3)	
Leukocyte, median (IQR), ×10^9^/L	9.24 (7.72, 11.08)	9.88 (8.23, 11.02)	9.03 (7.61, 11.26)	0.419
Neutrophil, median (IQR), ×10^9^/L	7.71 (5.82, 9.49)	8.06 (6.40, 9.87)	7.47 (5.50, 9.45)	0.326
Lymphocyte, median (IQR), ×10^9^/L	1.07 (0.82, 1.52)	1.06 (0.69, 1.52)	1.07 (0.83, 1.54)	0.677
Monocyte, median (IQR), ×10^9^/L	0.36 (0.29, 0.47)	0.33 (0.25, 0.43)	0.37 (0.30, 0.48)	0.061
NLR, median (IQR)	6.84 (4.30, 11.24)	8.72 (4.70, 11.60)	6.65 (4.14, 11.31)	0.331
Platelet, median (IQR), ×10^9^/L	205 (177, 245)	192 (158, 222)	208 (178, 248)	0.061
Albumin, median (IQR), g/L	40.39 (38.40, 43.03)	40.68 (37.29, 43.20)	40.30 (38.50, 43.00)	0.955
Prealbumin, median (IQR), mg/L	231 (189, 265)	238 (183, 268)	231 (189, 260)	0.998
Cholesterol, median (IQR), mmol/L	4.28 (3.70, 4.97)	4.13 (3.26, 5.17)	4.31 (3.74, 4.96)	0.723
Triglyceride, median (IQR), mmol/L	1.04 (0.74, 1.48)	1.12 (0.66, 1.68)	1.04 (0.76, 1.46)	0.790
HDL, median (IQR), mmol/L	1.17 (0.99, 1.44)	1.17 (0.97, 1.56)	1.17 (0.99, 1.43)	0.740
LDL, median (IQR), mmol/L	2.69 (2.13, 3.28)	2.60 (1.84, 3.37)	2.69 (2.15, 3.27)	0.681
Apoprotein A, median (IQR), g/L	1.23 (1.11, 1.35)	1.28 (1.17, 1.38)	1.21 (1.09, 1.33)	0.103
Apoprotein B, median (IQR), g/L	0.88 (0.74, 1.05)	0.89 (0.78, 1.02)	0.86 (0.74, 1.05)	0.644
Uric acid, median (IQR), μmol/L	334 (272, 400)	341 (268, 380)	331 (273, 402)	0.921
Urea, median (IQR), mmol/L	5.46 (4.38, 6.95)	6.76 (4.58, 7.82)	5.25 (4.31, 6.68)	0.052
Creatinine, median (IQR), μmol/L	63 (53, 73)	68 (54, 80)	62 (52, 70)	0.214
Admission RBG, median (IQR), mmol/L	6.96 (6.06, 8.60)	6.79 (6.46, 8.86)	6.99 (6.04, 8.45)	0.491
Post‐thrombectomy FBG, median (IQR), mmol/L	7.26 (6.04, 9.57)	8.45 (6.82, 12.47)	7.11 (5.91, 8.99)	**0.002** **
Fibrinogen, median (IQR), g/L	3.13 (2.62, 3.83)	3.26 (2.57, 3.74)	3.12 (2.63, 3.86)	0.939
D‐dimer, median (IQR), mg/L	0.80 (0.30, 2.56)	1.57 (0.42, 5.18)	0.70 (0.27, 2.13)	0.072
Intravenous thrombolysis, *n* (%)	41 (30.8)	12 (46.2)	29 (27.1)	0.059
OTP, median (IQR), min	514 (402, 755)	472 (380, 719)	522 (405, 765)	0.188
Procedure time, median (IQR), min	60 (36, 90)	74 (54, 104)	55 (34, 89)	0.062
Procedural technique, *n* (%)				
Stent retriever	11 (8.3)	2 (7.7)	9 (8.4)	0.445
Aspiration catheter	33 (24.8)	4 (15.4)	29 (27.1)	
Combined techniques	45 (33.8)	12 (46.2)	33 (30.8)	
Rescue angioplasty	44 (33.1)	8 (30.8)	36 (33.6)	
Number of passes, median (IQR)	2 (1, 3)	2 (1, 4)	2 (1, 3)	0.064
Successful reperfusion, *n* (%)	124 (93.2)	24 (92.3)	100 (93.5)	1.000
First pass recanalization, *n* (%)	43 (32.3)	7 (26.9)	36 (33.6)	0.511

Abbreviations: ASPECTS, Alberta Stroke Program Early CT Score; DBP, diastolic blood pressure; FBG, fasting blood glucose; HDL, high‐density lipoprotein cholesterol; ICA, internal carotid artery; LDL, low‐density lipoprotein cholesterol; M1, middle cerebral artery M1 segment; M2, middle cerebral artery M2 segment; NIHSS, National Institutes of Health Stroke Scale; OTP, onset‐to‐puncture time; RBG, random blood glucose; SBP, systolic blood pressure.

* *p* < 0.05.

** *p* < 0.01.

Parenchymal hematoma occurred in 26 of the 133 patients with large infarct cores (19.5%). Compared to the non‐PH patients, patients who developed PH showed a higher prevalence of coronary artery disease (46.2% vs. 26.2%, *p* = 0.046), higher baseline NIHSS scores (18 vs. 15, *p* = 0.026), lower baseline ASPECTS (3 vs. 4, *p* = 0.007), and higher post‐thrombectomy fasting blood glucose levels (8.45 vs. 7.11 mmol/L, *p* = 0.002). In addition, the PH group demonstrated trends toward lower monocyte (*p* = 0.061), lower platelet (*p* = 0.061), higher urea (*p* = 0.052), higher D‐dimer (*p* = 0.072), a higher rate of IVT (*p* = 0.059), longer procedure time (*p* = 0.062), and a greater number of passes (*p* = 0.064), though these differences were not statistically significant.

### 3.2 Variable Selection via Lasso‐Logistic Analysis

**FIGURE 2:**
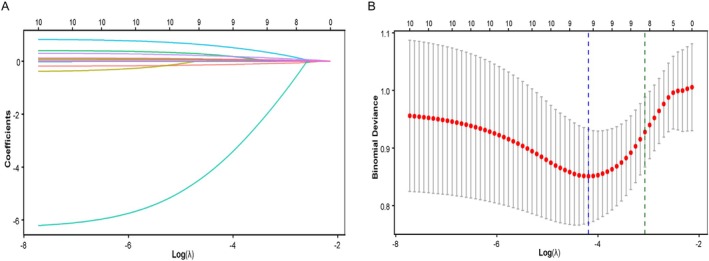
LASSO regression for variable selection. (A) LASSO coefficient profiles. Trajectories of coefficients for candidate variables are shown relative to the log (*λ*) sequence. (B) Tuning parameter selection. The optimal log (*λ*) was identified by calculating the binomial deviance through 5‐fold cross‐validation. Vertical dashed lines mark the *λ* values corresponding to the minimum deviance (*λ*_min_) and the 1‐standard error criteria (*λ*_1se_).

**FIGURE 3:**
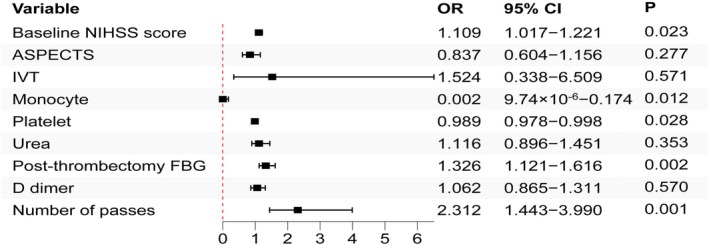
Identification of independent predictors for PH via multivariable logistic regression analysis. FBG, fasting blood glucose.

By LASSO regression with five‐fold cross‐validation at λ.min, nine variables were selected for PH prediction out of 11 variables discovered via univariable analysis (*p* < 0.1) (Figure 2A,B). These nine variables, including baseline NIHSS score, ASPECTS, IVT, monocytes, platelets, urea, post‐thrombectomy FBG, D‐dimer, and number of passes, were further analyzed with a multivariable logistic regression. After adjusting for the other model covariates, baseline NIHSS score (OR 1.109, 95% CI 1.017–1.221; *p* = 0.023), monocytes (OR 0.002, 95% CI 9.74 × 10^−6^–0.174; *p* = 0.012), platelets (OR 0.989, 95% CI 0.978–0.998; *p* = 0.028), post‐thrombectomy FBG (OR 1.326, 95% CI 1.121–1.616; *p* = 0.002), and number of passes (OR 2.312, 95% CI 1.443–3.990; *p* = 0.001) were identified as independent predictors of PH following EVT in patients with large core infarctions (Figure 3).

### 3.3 Prediction Model Construction and Evaluation

**FIGURE 4:**
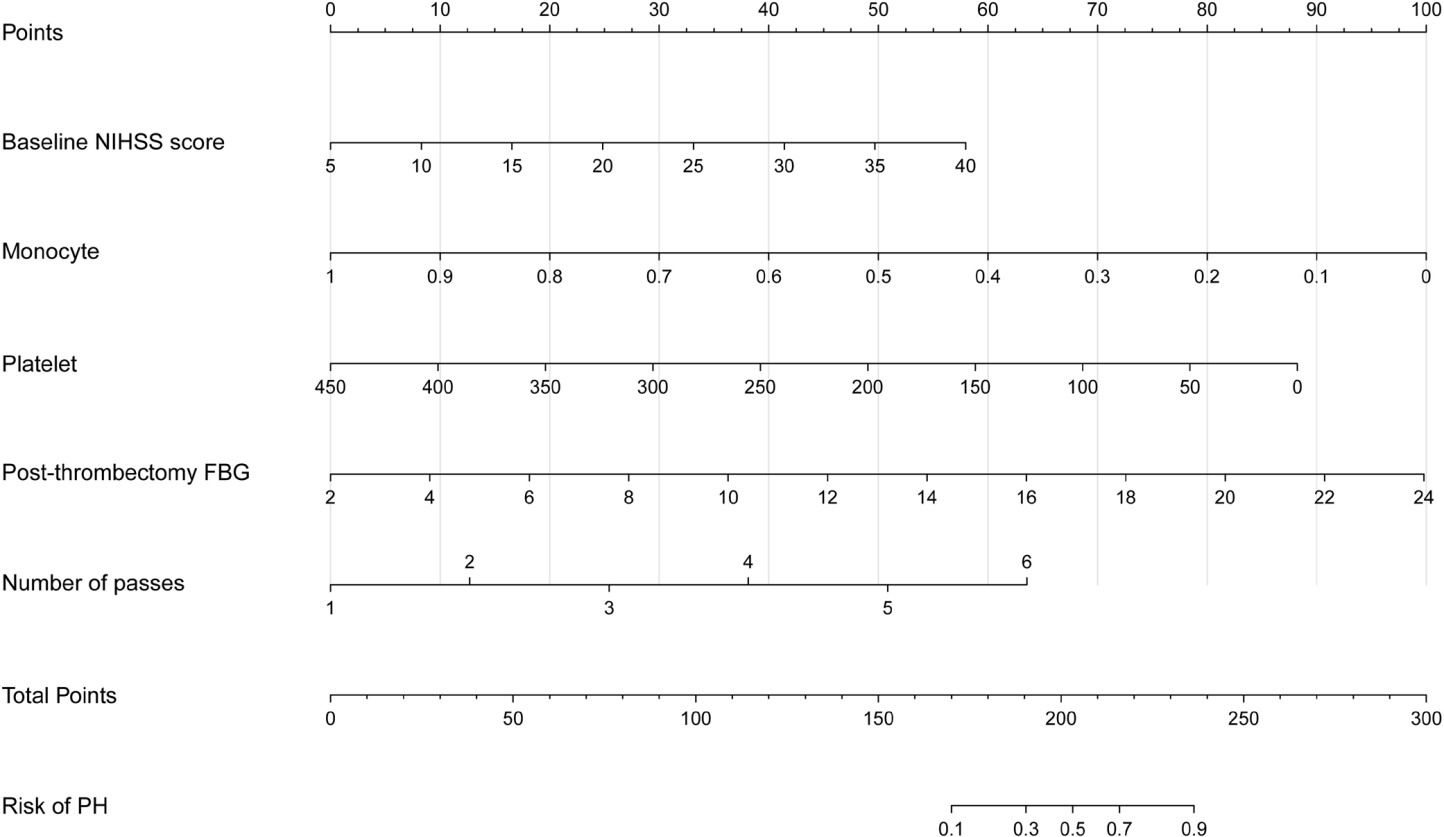
The nomogram model for predicting PH after EVT in patients with large core infarctions.

**FIGURE 5:**
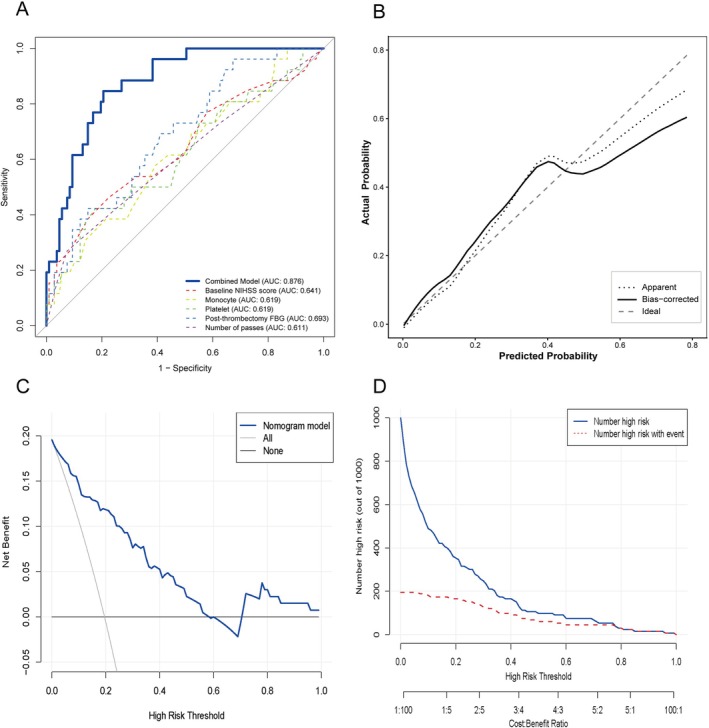
Evaluation of the nomogram model. (A) Receiver operating characteristic (ROC) curves of the nomogram model and predictive factors. (B) Calibration curve of the nomogram model. (C) Decision curve analysis (DCA) of the nomogram model. (D) Clinical impact curve of the nomogram model.

A nomogram was established to estimate the risk of PH using five independently associated predictors: baseline NIHSS score, monocytes, platelets, post‐thrombectomy FBG, and the number of passes (Figure 4). The model exhibited good discriminative capacity with an AUC of 0.876 (95% CI: 0.812–0.940), which was significantly superior to any single predictor (Figure 5A). Specifically, the individual AUC values were 0.641 for baseline NIHSS, 0.619 for monocytes, 0.619 for platelets, 0.693 for post‐thrombectomy FBG, and 0.611 for the number of passes. The calibration curve showed close alignment with the ideal reference line, indicating acceptable agreement between the nomogram‐estimated probabilities and the observed incidence of PH (Figure 5B). This satisfactory calibration was also supported by a non‐significant Hosmer‐Lemeshow test (*χ* ^2^ = 3.097, *p* = 0.928) and a low Brier score of 0.111. Regarding clinical utility, DCA showed that the nomogram achieved higher net benefit compared with the treat‐all and treat‐none approaches over varying threshold probabilities among patients undergoing EVT (Figure 5C). The clinical impact curve (Figure 5D) further validated its practical value. Notably, at threshold probabilities exceeding 0.4, the estimated number of high‐risk people closely matched the actual observed PH events, indicating high specificity within this range. Internal validation using bootstrap resampling confirmed the robustness and reliability of the PH prediction model, with a bias‐corrected AUC of 0.844 (95% CI: 0.777–0.914) and a corrected Brier score of 0.128.

### 3.4 Subgroup Analyses of PH Risk

Within subgroups stratified by age (≥ 70 vs. < 70 years), baseline ASPECTS (0–2 vs. 3–5), and IVT status (yes vs. no), Table S1 shows the distribution of these variables between patients with and without PH. In the younger subgroup (age < 70), patients with PH exhibited significantly lower monocyte counts (0.31 vs. 0.40, *p* = 0.045) and higher post‐thrombectomy FBG levels (8.47 vs. 6.72, *p* = 0.006) than non‐PH patients; however, these differences were not observed in the elderly subgroup. In the ASPECTS 3–5 subgroup, patients with PH showed higher baseline NIHSS scores (19 vs. 15, *p* = 0.044) and lower monocyte counts (0.28 vs. 0.36, *p* = 0.029) than non‐PH patients; whereas in the ASPECTS 0–2 subgroup, only post‐thrombectomy FBG differed significantly between groups, with higher values observed in patients with PH (8.95 vs. 7.96, *p* = 0.044). For patients receiving bridging IVT therapy, PH patients had markedly higher post‐thrombectomy FBG (12.13 vs. 6.91, *p* = 0.002), a difference not seen in the non‐IVT group. Conversely, among patients without IVT, PH was associated with a higher number of passes (3 vs. 2, *p* = 0.003).

**FIGURE 6:**
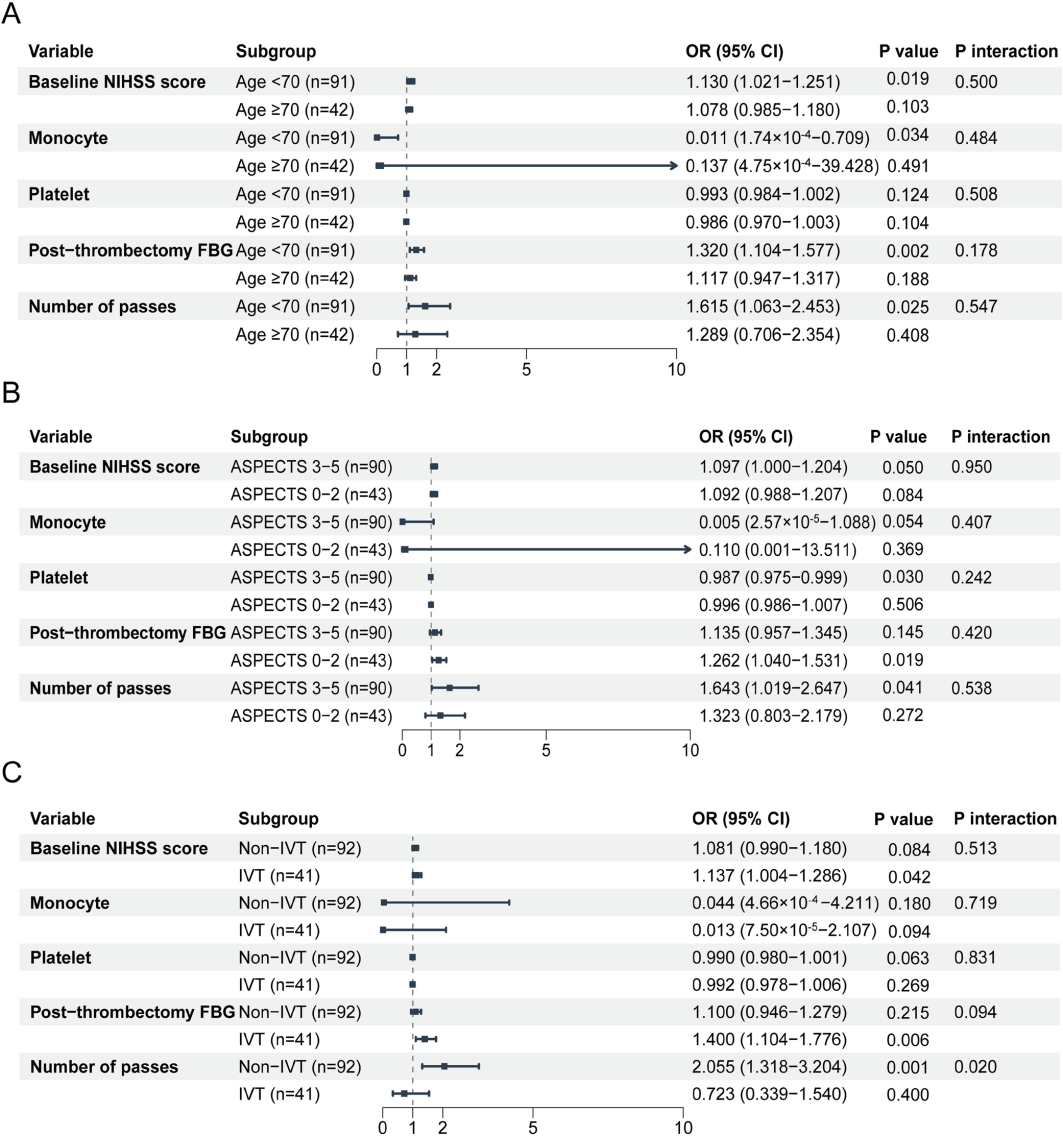
Forest plots of univariable logistic regression for predictors of PH across clinical subgroups. Subgroup analyses were stratified by (A) age (< 70 vs. ≥ 70 years), (B) baseline ASPECTS (3–5 vs. 0–2), and (C) pre‐procedural intravenous thrombolysis (Non‐IVT vs. IVT).

Univariable logistic regression across subgroups (Figure 6) further revealed that post‐thrombectomy FBG was significantly associated with PH in the ASPECTS 0–2 (OR 1.262, 95% CI 1.040–1.531; *p* = 0.019), age < 70 (OR 1.320, 95% CI 1.104–1.577; *p* = 0.002), and IVT subgroups (OR 1.400, 95% CI 1.104–1.776; *p* = 0.006). However, the interaction tests were not statistically significant, providing no evidence that the association between post‐thrombectomy FBG and PH differed according to baseline ASPECTS, age, or IVT status. Notably, the number of passes significantly increased PH risk in the non‐IVT group (OR 2.055, 95% CI 1.318–3.204; *p* = 0.001) but not in the IVT group. The unadjusted logistic regression further revealed a significant interaction between the number of passes and IVT (*p* for interaction = 0.020). This suggests that the procedural burden of multiple passes may specifically elevate PH risk in patients without prior bridging thrombolysis. No other significant interactions were observed between predictors and age, ASPECTS, or IVT (all *p* for interaction > 0.05).

### 3.5 PH and 90‐Day Prognosis

In unadjusted analyses, PH was associated with poorer clinical outcomes at 90 days. Patients who developed PH exhibited a higher mRS score (5 vs. 3, *p* < 0.001) and were less likely to achieve functional independence (mRS 0–2: 3.8% vs. 38.3%, *p* < 0.001) and independent ambulation (mRS 0–3: 15.4% vs. 51.4%, *p* < 0.001) than those without PH (Figure S1). In addition, the patients with PH demonstrated a higher rate of 90‐day mortality than the non‐PH population (38.5% vs. 11.2%, *p* = 0.002). In the multivariable logistic regression model adjusted for age, sex, baseline NIHSS score, baseline ASPECTS, number of passes, and successful reperfusion, PH still showed an independent association with decreased odds of functional independence at 90 days (OR, 0.107; 95% CI, 0.012–0.933; *p* = 0.043). By contrast, the associations of PH with independent ambulation (OR, 0.312; 95% CI, 0.086–1.135; *p* = 0.077) and 90‐day mortality (OR, 3.517; 95% CI, 0.853–14.490; *p* = 0.082) were not statistically significant, despite OR estimates suggesting a potential trend toward worse outcomes.

## 4 Discussion

Among patients with large core infarctions receiving EVT, we identified clinical factors associated with PH and developed a predictive nomogram. The incidence of PH was 19.5%, consistent with the previously reported range of 16.1%–29.9% [9, 10]. PH had an association with a decreased probability of achieving functional independence at 90 days. Therefore, identifying patients susceptible to PH at an early stage is critical for optimizing clinical management. We established a nomogram incorporating baseline NIHSS scores, monocytes, platelets, post‐thrombectomy FBG and the number of passes and internally validated the model. The final model demonstrated good predictive performance and calibration, filling a critical gap in early PH risk stratification for this population. Furthermore, subgroup analyses revealed a significant interaction between the number of passes and IVT. Among patients not receiving bridging thrombolysis, more passes increased PH risk, while no significant relationship was identified in those undergoing IVT.

Prior data have indicated independent associations of PH after thrombectomy with poorer functional prognosis and elevated mortality in patients with large core infarcts [9]. Our analysis revealed that PH reduced the likelihood of functional independence at 90 days, whereas its associations with independent ambulation and mortality did not reach statistical significance. Several reasons may account for this discrepancy. First, the severe baseline neurological deficits and extensive ischemic injury inherent to this population may be major determinants of disability and mortality, thereby attenuating the additional effect of PH. Second, the modest sample size and limited occurrence of outcome events might have constrained the statistical ability to identify a significant association between PH and mortality or independent ambulation after adjusting for major confounders. Nevertheless, the marked reduction in the likelihood of functional independence associated with PH highlights its contribution to disability burden and underscores the importance of early detection of risky patients to facilitate preventive and management strategies.

Our study demonstrated that baseline NIHSS score, monocytes, platelets, post‐thrombectomy FBG, and number of passes are predictive factors of PH following EVT in large core infarction. Baseline NIHSS is a described independent risk factor of blood–brain barrier (BBB) disruption in AIS [12]. Higher baseline NIHSS scores have been reported to exacerbate the risk of post‐thrombectomy hemorrhagic complications including sICH [13, 14], as well as HI and PH [13]. Our results reinforce the value of stroke severity in predicting and stratifying PH risk specifically for large core infarctions. Regarding monocytes, their role in post‐stroke BBB integrity is dualistic: while early‐phase monocytes may impair the BBB, M2‐type macrophages often exert vasculoprotective effects through inflammatory modulation [15]. Consistent with our results, previous research has indicated that lower serum monocyte counts are independently associated with the development of HT among patients receiving EVT [16]. Furthermore, a study has shown that higher enrichment of monocytes/macrophages in thrombi is related to better functional prognosis and decreased risk of PH [17]. Collectively, these observations may reflect a potential vasculoprotective involvement of monocytes after ischemia–reperfusion. Future longitudinal research is needed to elucidate the temporal dynamics of monocyte subsets and their mechanistic contribution to BBB injury. Conversely, thrombocytopenia serves as a risk factor for any intracranial hemorrhage after EVT and is linked to increased mortality [18]. Our data further corroborate that lower platelet counts elevate the risk of PH in large territorial cerebral infarction.

In our study, post‐thrombectomy FBG, rather than baseline glucose, independently predicted PH, reflecting the dynamic nature of acute‐phase glycemia. While admission hyperglycemia may be transient and stress‐induced, post‐procedural FBG may indicate sustained metabolic disturbance and secondary injury during post‐recanalization BBB compromise. Mechanistically, hyperglycemia amplifies oxidative stress and inflammatory cascades, thereby exacerbating BBB disruption and hemorrhagic transformation following reperfusion [19, 20]. An analysis based on the RESCUE‐Japan LIMIT trial also demonstrated that patients experiencing transient, delayed, or persistent severe hyperglycemia during treatment showed a lower probability of achieving good functional recovery (mRS 0–3) and had a higher risk of sICH than those without severe hyperglycemia [21]. These findings advocate for shifting clinical priority toward post‐procedural glycemic management. While current guidelines endorse managing hyperglycemia, intensive targets (80–130 mg/dL) are discouraged due to hypoglycemia risks without functional benefit [22]. Larger prospective cohort studies and clinical trials are required in the future to determine optimal glycemic goals in individuals with large core infarctions.

This study identified the number of thrombectomy passes as an independent predictor associated with PH post‐EVT. This finding aligns with the post hoc analysis of the ANGEL‐ASPECT trial, which indicated a positive association between increased attempts and PH in large‐core infarction patients [9]. Similarly, a multicenter retrospective analysis involving AIS patients with ASPECTS 3–5 treated with EVT suggested that patients with > 2 tries of recanalization had limited functional benefit and increased frequency of sICH and mortality [23]. Secondary analysis of the RESCUE‐Japan LIMIT study further indicated that intracranial hemorrhage incidence during 48 h was significantly higher following ≥ 2 passes compared with medical management alone [24]. The increased number of passes may be attributed to two underlying factors: ICA tortuosity, which reduces first‐pass recanalization effect and necessitates repeated attempts [25], and fibrin‐rich thrombus composition, which is inherently more resistant to recanalization and associated with a greater number of procedural passes [26]. These findings underscore the need for preoperative assessment frameworks characterizing ICA tortuosity and thrombus composition to identify high‐risk patients and optimize thrombectomy strategies.

Subgroup analyses identified a significant interaction between the number of passes and IVT on PH risk: increasing passes was associated with elevated PH risk exclusively in patients without bridging IVT, suggesting that IVT may reduce and soften thrombi and reduce endothelial trauma during repeated retrieval [27, 28, 29], whereas untreated thrombi are more cohesive and adherent, amplifying mechanical vascular injury. This observation highlights the potential importance of minimizing unnecessary repeat thrombectomy passes, particularly in patients undergoing direct thrombectomy. Nevertheless, these findings need to be viewed with caution, considering the modest number of cases and the exploratory nature of this uncorrected interaction assessment. Further prospective research is necessary to investigate whether the association of thrombectomy passes and PH differs by IVT status in large core infarctions. By contrast, although exploratory subgroup analyses suggested post‐thrombectomy FBG was significantly associated with PH within the ASPECTS 0–2, age < 70 years, and bridging IVT subgroups, the interaction tests did not reach statistical significance. Therefore, these differences in statistical significance may reflect variations in sample size and statistical power rather than true differences in effect.

The predictive model developed in this study offers several notable advantages. First, unlike previously established models for HT or PH in general EVT populations [30, 31, 32], our model specifically targets patients with large core infarctions, thereby addressing a critical gap in PH prediction for this high‐risk subgroup. Previous studies mainly identified individual predictors, including alcohol use, number of thrombectomy attempts, and local sedative anesthesia [9, 10], without constructing an integrated risk‐prediction tool for PH after EVT in large core ischemic stroke. Second, by incorporating readily accessible clinical and laboratory parameters, the model demonstrates promising discrimination and calibration, ensuring high clinical utility. Using the nomogram, clinicians can detect patients at increased risk of PH in the early phase and implement individualized management strategies, including more stringent blood pressure control, intensified neurological monitoring, timely neuroimaging reassessment, avoidance of early anticoagulation, rigorous glycemic management, and neuroprotective interventions. Such targeted measures may help mitigate the likelihood of PH and potentially improve functional recovery among patients with large core infarctions.

Several limitations of the present study warrant consideration when interpreting the findings. First, the retrospective characteristic of this study and single‐center setting may have resulted in potential selection bias and restricted the applicability of our observations to larger populations. Second, the relatively modest sample size may have constrained our ability to detect additional predictors and limited the precision of subgroup analyses. Moreover, the interaction analysis was performed using an unadjusted model due to the small number of outcome events across subgroups. The observed interaction can be viewed as exploratory and warrants further validation in larger, powered cohorts. Third, despite incorporating several covariates in the multivariable analysis, unknown factors not included in the analysis may affect the estimated associations. Finally, while our nomogram demonstrated promising performance through internal validation, the absence of external validation suggests these estimates may be optimistic. Future studies using independent multicenter prospective cohorts are needed to assess whether the nomogram maintains its predictive ability in different clinical settings and populations.

## 5 Conclusions

This study established and internally validated a PH prediction nomogram incorporating baseline NIHSS score, monocytes, platelets, post‐thrombectomy FBG, and the number of passes in patients with large core anterior circulation infarctions receiving EVT. The model demonstrated good predictive performance and may facilitate identification of patients vulnerable to PH during the early phase, thereby supporting personalized management strategies following EVT. Notably, the interaction between number of passes and IVT underscores the complexity of PH pathogenesis in large core infarctions. Further validation through multicenter prospective cohorts is required to ensure the predictive stability and broader generalizability of the proposed nomogram.

## Author Contributions

Jiamin Li: writing – original draft, methodology, investigation, software, formal analysis, data curation, conceptualization. Zixin Wang: writing – original draft, methodology, investigation, software, formal analysis, data curation, conceptualization. Xinyi Wang: writing – review and editing, software, methodology, formal analysis. Boyi Yuan: writing – review and editing, investigation, visualization. Yun Chen: writing – review and editing, investigation, visualization. Wan Wang: writing – review and editing, validation, visualization. Jiameng Li: writing – review and editing, investigation. Jiapeng Zhao: writing – review and editing, investigation. Jinming Sheng: writing – review and editing. Qingfeng Ma: review and editing, resources, supervision, project administration, funding acquisition, conceptualization.

## Funding

This study was funded by the National Natural Science Foundation of China (82271312).

## Ethics Statement

The study adhered to the Declaration of Helsinki and was approved by the Ethics Committee of Xuanwu Hospital, Capital Medical University (approval no. [2021]008). In accordance with institutional requirements, informed consent was waived for the study's retrospective nature.

## Conflicts of Interest

The authors declare no conflicts of interest.

## Supporting information


**Table S1:** Distribution of variables in patients with versus without PH across subgroups of age (≥ 70 vs. < 70 years), baseline ASPECTS (0–2 vs. 3–5), and intravenous thrombolysis (IVT vs. Non‐IVT).
**Figure S1:** Distribution of mRS scores of PH group and non‐PH group.

## Data Availability

The data that support the findings of this study are available on request from the corresponding author. The data are not publicly available due to privacy or ethical restrictions.
