# Supplementary material for: Predictors and a Nomogram for Parenchymal Hematoma After Endovascular Thrombectomy in Large Core Ischemic Stroke
Source: CNS Neurosci Ther. 2026 Aug 13;32(8):e71087. doi: 10.1002/cns.71087 (PMC13470228; doi:10.1002/cns.71087)
Supplement: Supplementary file 1 [file CNS-32-e71087-s001.docx]

**Table S1. Distribution of variables in patients with versus without PH across subgroups of age (≥70 vs <70 years), baseline ASPECTS (0–2 vs 3–5), and intravenous thrombolysis (IVT vs Non-IVT)**

| **Subgroup** | **Variable** | **PH** | **Non-PH** | **P value** |
| --- | --- | --- | --- | --- |
| Age <70 (n=91) | Baseline NIHSS score | 18 (13-21) (n=17) | 16 (12-18) (n=74) | 0.060 |
|  | Monocyte | 0.31 (0.25-0.43) (n=17) | 0.40 (0.31-0.49) (n=74) | **0.045** |
|  | Platelet | 202 (163-244) (n=17) | 209 (182-260) (n=74) | 0.287 |
|  | Post thrombectomy FBG | 8.47 (6.91-12.31) (n=17) | 6.72 (5.72-8.76) (n=74) | **0.006** |
|  | Number of passes | 2 (2-4) (n=17) | 2 (1-2) (n=74) | 0.065 |
| Age ≥70 (n=42) | Baseline NIHSS score | 16 (15-20) (n=9) | 14 (13-19) (n=33) | 0.156 |
|  | Monocyte | 0.34 (0.32-0.37) (n=9) | 0.35 (0.28-0.43) (n=33) | 0.842 |
|  | Platelet | 171 (149-209) (n=9) | 198(174-233) (n=33) | 0.107 |
|  | Post thrombectomy FBG | 8.19 (7.56-12.16) (n=9) | 7.42 (6.47-11.04) (n=33) | 0.177 |
|  | Number of passes | 2 (1-3) (n=9) | 2(1-3) (n=33) | 0.528 |
| ASPECTS 3-5 (n=90) | Baseline NIHSS score | 19 (16-20) (n=13) | 15 (12-18) (n=77) | **0.044** |
|  | Monocyte | 0.28 (0.25-0.35) (n=13) | 0.36 (0.30-0.49) (n=77) | **0.029** |
|  | Platelet | 182 (161-220) (n=13) | 209 (181-248) (n=77) | 0.067 |
|  | Post thrombectomy FBG | 8.19 (6.55-11.30) (n=13) | 6.91 (5.89-8.99) (n=77) | 0.052 |
|  | Number of passes | 2 (2-3) (n=13) | 2 (1-3) (n=77) | 0.100 |
| ASPECTS 0-2 (n=43) | Baseline NIHSS score | 15 (14-23) (n=13) | 17 (13-18) (n=30) | 0.442 |
|  | Monocyte | 0.37 (0.31-0.44) (n=13) | 0.38 (0.28-0.46) (n=30) | 0.711 |
|  | Platelet | 199 (163-212) (n=13) | 198 (174-236) (n=30) | 0.625 |
|  | Post thrombectomy FBG | 8.95 (7.56-16.27) (n=13) | 7.96 (6.43-8.86) (n=30) | **0.044** |
|  | Number of passes | 2 (1-4) (n=13) | 2 (1-2) (n=30) | 0.419 |
| Non-IVT (n=92) | Baseline NIHSS score | 19 (15-20) (n=14) | 15 (12-18) (n=78) | 0.064 |
|  | Monocyte | 0.32 (0.26-0.40) (n=14) | 0.36 (0.30-0.48) (n=78) | 0.213 |
|  | Platelet | 167 (152-225) (n=14) | 207 (179-253) (n=78) | 0.084 |
|  | Post thrombectomy FBG | 7.46 (6.54-8.37) (n=14) | 7.12 (5.80-8.85) (n=78) | 0.254 |
|  | Number of passes | 3 (2-5) (n=14) | 2 (1-2) (n=78) | **0.003** |
| IVT (n=41) | Baseline NIHSS score | 16 (14-26) (n=12) | 16 (12-18) (n=29) | 0.200 |
|  | Monocyte | 0.32 (0.24-0.43) (n=12) | 0.39 (0.30-0.47) (n=29) | 0.192 |
|  | Platelet | 201 (178-214) (n=12) | 208 (177-237) (n=29) | 0.448 |
|  | Post thrombectomy FBG | 12.13 (8.83-13.77) (n=12) | 6.91 (6.30-9.04) (n=29) | **0.002** |
|  | Number of passes | 2 (1-2) (n=12) | 2 (2-3) (n=29) | 0.366 |

FBG, fasting blood glucose; IVT, intravenous thrombolysis.

**Figure S1. Distribution of mRS scores of PH group and non-PH group.**
